# Establishing the effectiveness of technology-enabled dementia education for health and social care practitioners: a systematic review

**DOI:** 10.1186/s13643-021-01781-8

**Published:** 2021-09-21

**Authors:** Kevin Muirhead, Leah Macaden, Keith Smyth, Colin Chandler, Charlotte Clarke, Rob Polson, Chris O’Malley

**Affiliations:** 1 Department of Nursing & Midwifery, School of Health, Social Care & Life Sciences, University of the Highlands and Islands, Centre for Health Science, Old Perth Road, Inverness, IV2 3JH UK; 2 Learning and Teaching Academy, University of the Highlands and Islands, An Lòchran, Inverness Campus, Inverness, IV2 5NA UK; 3 School of Health in Social Science, University of Edinburgh, Buccleuch Place, Edinburgh, EH8 9LN UK; 4 Faculty of Social Sciences and Health, Durham University, Arthur Holmes Building, Lower Mountjoy, South Road, Durham, DH1 3LE UK; 5 Highland Health Sciences Library, Centre for Health Science, Old Perth Road, Inverness, IV2 3JH UK

**Keywords:** Dementia, Dementia education, Dementia training, Technology-enabled learning, Systematic review, Effectiveness

## Abstract

**Background:**

Dementia prevalence is increasing globally and yet evidence suggest that gaps exist in dementia-specific knowledge among health and social care practitioners. Technological modes of educational delivery may be as effective as traditional education and can provide practitioners with increased accessibility to dementia training. Benefits of digitally based dementia education have been established including pedagogical strategies that influence dementia knowledge and care attitudes. This review aimed to appraise and synthesise contemporary experimental evidence that evaluated technology-enabled dementia education for health and social care practitioners. Outcomes based on Kirkpatrick’s Model were learner satisfaction; knowledge, skills, and attitudes; behaviours; and results.

**Methods:**

MEDLINE, CINAHL, and Web of Science were among 8 bibliographic databases searched from January 2005 until February 2020. Keywords included dementia and education (and terms for technological modes of education, learning, or training). We included experimental and quasi-experimental studies. Medical Education Research Study Quality Instrument established the overall quality of included studies and pragmatic application of Mixed Methods Appraisal Tool established individual study quality and highlighted methodological features of educational research. Narrative synthesis was conducted as heterogeneous outcome data precluded meta-analysis.

**Results:**

We identified 21 relevant studies: 16 evaluated online dementia education and 5 evaluated computer-based approaches. Most studies used before-after designs and study quality was moderate overall. Most studies reported knowledge-based outcomes with statistically significant findings favouring the training interventions. Positive effects were also observed in studies measuring skills and attitudinal change. Fewer studies reported significant findings for behavioural change and results due to training. Case-based instruction was a frequently described instructional strategy in online dementia education and videos were common information delivery modes. CD-ROM training and simulation activities were described in computer-based dementia education.

**Discussion:**

Future emphasis must be placed on teaching and learning methods within technology-enabled dementia education which should be role relevant and incorporate active and interactive learning strategies. Future evaluations will require contextually relevant research methodologies with capacity to address challenges presented by these complex educational programmes and multi-component characteristics.

**Systematic review registration:**

This systematic review is based on a protocol registered with PROSPERO (CRD42018115378).

**Supplementary Information:**

The online version contains supplementary material available at 10.1186/s13643-021-01781-8.

## Background

Dementia is a global public health priority and significant challenge for health and social care [1, 2]. Fifty million people are estimated to be living with dementia globally and the prevalence is anticipated to rise to 152 million by 2050 [3]. Within the UK, 850,000 people (1 in 14 adults over the age of 65) are estimated to be living with dementia and future prevalence is predicted to mirror global trends [4]. In Scotland, dementia has been a national priority since 2007 [5], prompting a series of national strategies for better dementia care and services [6–8]. Concern about the quality of care for people living with dementia has intensified the need for an appropriately educated workforce [9] with evidence suggesting gaps in dementia-specific knowledge among practitioners [10].

The dementia care setting is comprised of health and social care services that are delivered within hospitals, primary care, residential, and nursing homes, as well as community care [11]. Dementia education is required in all care contexts. Skilled, knowledgeable, and dementia competent staff are critical for person-centred dementia care in the acute hospital [12]. Hospital environments can be unsuitable for dementia care and inadequate staff training can result in unmet care needs and an increase in behavioural and non-cognitive symptoms of dementia—which staff report to be burdensome [13]. In primary care, dementia education is particularly helpful to support early diagnosis, appropriate treatment, and on-going support [14, 15]. However, the primary care workforce has had limited access to dementia education and a range of training needs have been identified [16]. Inadequate caregiver training in social care environments including care homes can result in low-level staff morale and staff retention difficulties that negatively impact care quality [17]. Furthermore, it is essential that the early clinical experiences of health and social care practitioners (HSCPs) is underpinned by clear and relevant undergraduate dementia education that is related to the knowledge, skills, and attitudes that are required to care effectively for people living with dementia [18].

**Fig. 1:**
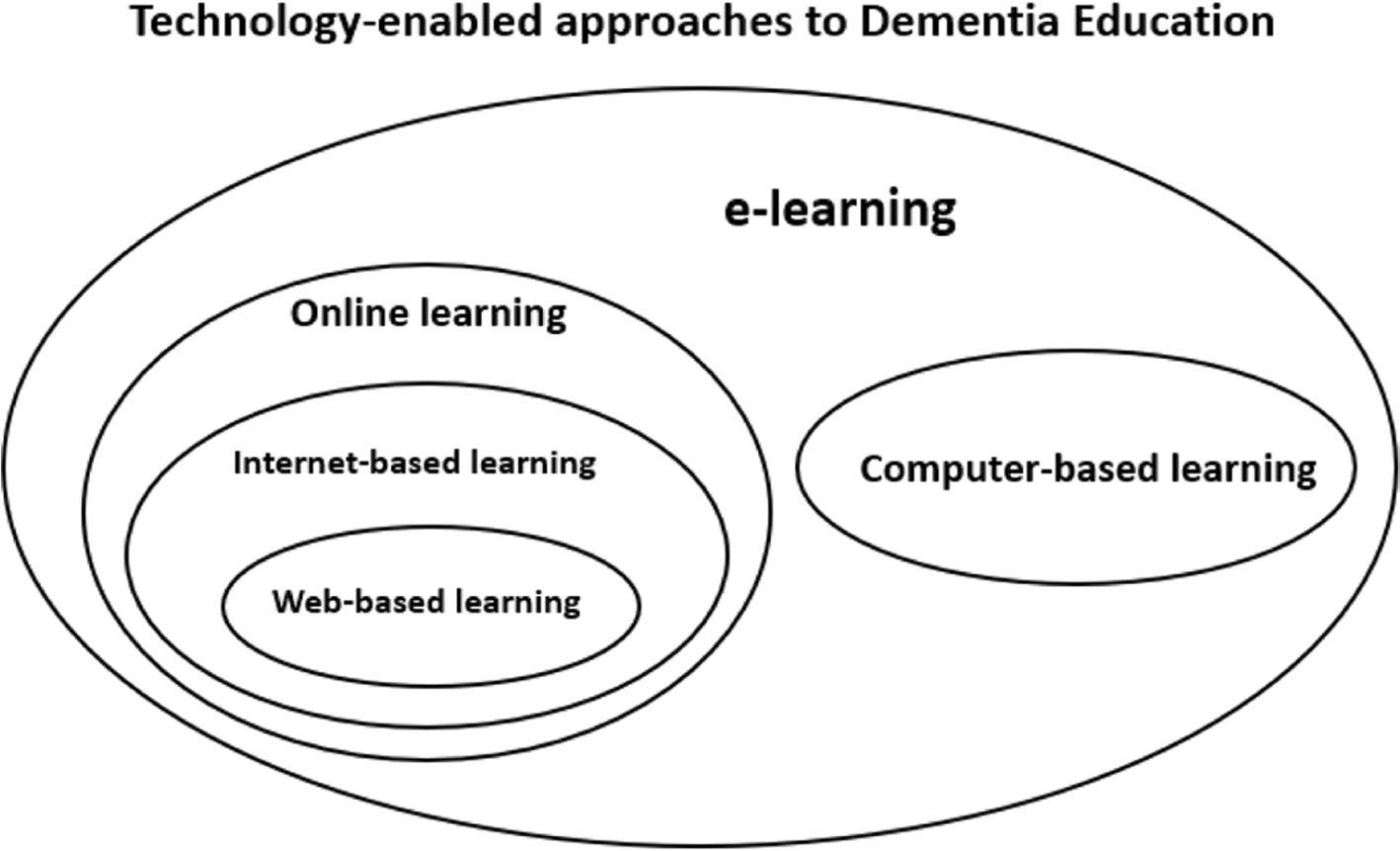
Relationship of key terms in technology-enabled dementia education. Adapted [20]

Technology-enabled dementia education (TEDE) may provide learning opportunities for HSCPs through increased accessibility to dementia training in the multiple practice contexts. TEDE refers to a collection of methods that use the application of some form of digital technology for teaching and/or learning in the dementia education context. The word *enabled* refers to facilitation: dementia education is made possible by the use of technology (definition adapted [19]). TEDE programmes encompass several subsets of delivery methods not limited to e-learning, online learning, and other computer-based learning modalities including blended methods that integrate face-to-face teaching and learning with technological approaches. These delivery methods have their own nuance of meaning and subset relationships exist between them (Fig. 1). The concept of internet-based learning, for example, is broader than web-based learning since the web is only one of many internet services. Online learning can be arranged through many networks, so, internet-based learning is only a subset of online learning. Computer-based learning implies that the computer is not connected to a network; therefore, computer-based learning is not a subset of online learning. E-learning can take place via any electronic medium, so, online learning and computer-based (non-networked) learning are both subsets of e-learning [20]. Definitions for the key terms are shown in Table 1. The definitions highlight that subset terms may be, and often are, used interchangeably which is a potential source of confusion. Furthermore, the pedagogical aspect of the term (e.g., learning) may be replaced with synonyms such as education/instruction/training. In the TEDE context, the pedagogical aspect may be specified further (e.g., dementia education).

**Table 1:** Definitions of key terms in technology-enabled dementia education

**Term**	**Definition**
TEDE	A collection of methods that use the application of some form of digital technology for teaching and/or learning in the dementia education context.
E-learning	Learning via any electronic medium. E-learning refers to the application of information communication technology in its widest sense to support and improve the learning experience. Online and non-networked computer-based learning are subsets of e-learning.
Online learning	Online learning refers to any e-learning that is conducted online. Internet-based learning and web-based learning are subsets of online learning. The terms are often used interchangeably.
Computer-based learning	This is a non-networked approach for e-learning that emphasises the use of a computer (or computerised device) as the delivery platform.
Blended learning	A mixed mode of delivery combining face-to-face learning with e-learning techniques. It is especially relevant to introducing elements of flexibility into traditional courses.

The definitions were formulated for the review context and adapted from relevant sources [19–21]

*TEDE* Technology-enabled dementia education

E-learning has gained popularity due to cost effectiveness, high flexibility, and reduced dependence on geographical boundaries [22]. Evidence suggests that it is as effective, and possibly superior to traditional learning for undergraduate health professionals [23]. Among licensed healthcare practitioners, e-learning is associated with no important benefits compared to traditional learning [22]; however, the relative efficacy of e-learning must also factor critical dimensions relating to accessibility and acceptability. In a review of dementia training programmes for staff working in general hospital settings, Scerri et al. [24] reported on a study where the uptake of a self-directed dementia related e-learning programme among nurses was poor. The review suggested that e-learning may not be feasible in the acute hospital setting due to limitations in participants’ time, internet access, and digital competence. Surr and Gates [25] reported on the same study where 26 staff signed up to undertake the training but only six people completed it. Indeed, all participating staff chose to complete the education modules in hard copy rather than online [26]. Furthermore, Surr et al. [27] reported completion rates from a study of online dementia education being only 50%. These findings are not consistent across all reviews of dementia education. For instance, Elliot et al. [11] reported that satisfaction and compliance to attend dementia training was higher among nurses who completed training using a computer resource compared to nurses who attended traditional group training in a lecture format.

There is evidence to suggest that the flexibility of e-learning can be beneficial [27]; however, Surr and Gates [25] highlighted that approaches that rely on individuals to schedule their own time for dementia training, including e-learning, may lead to poorer outcomes in terms of knowledge gains and attitudinal change. The evidence that e-learning can contribute directly to these learning outcomes is mixed and may be dependent upon more nuanced pedagogical methods. Surr et al. [27] reported that confidence, competence, and self-efficacy were achieved following *interactive* web-based resources with evidence suggesting that *non-interactive* approaches may be less effective. In general, active learning, for example, using online multimedia was considered to be more effective than passive approaches such as watching an online video lecture. Where e-learning was utilized, learners preferred a combination of individual study with opportunities for online or face-to-face discussion. Online discussions were felt to be particularly beneficial to learning; however, time demands for learners and facilitators and the need for specialist technical support suggested this as a resource intensive form of study [27].

Scerbe et al. [28] conducted a review that focused exclusively on digital tools for the delivery of dementia education for health-care providers. The review included 10 studies that used pre- and post-test measures of evaluation. The teaching and learning methods detailed within studies included videos, audio-narration, graphics, and some interactive content including discussion forums. The review established that all of the included studies demonstrated a positive change on the outcomes measured and the review concluded there was compelling confirmation of effectiveness for digitally conveyed dementia education. Scerri et al. [24] highlighted that the heterogeneity of dementia training programmes can make it difficult to determine whether outcomes can be attributed to the interventions. Therefore, methods to evaluate TEDE may require capacity to demonstrate outcomes based on specific delivery methods and also the more nuanced teaching and learning methods contained within training programmes.

The pace of technological progress also requires consideration as this may influence pedagogical practices and subsequent learning outcomes. Web 2.0, for instance, resulted in a paradigm shift for teaching and learning online. Adopted in popular commentary in 2005 [29], Web 2.0 describes the transformation of the static ‘read only’ Web 1.0 into a dynamic ‘read-and-write’ participatory media [30]. This has facilitated interconnectivity and the interactive learning opportunities which are likely to be valuable when using technology for dementia education [27]. Therefore, contemporary TEDE programmes may have additional capacity to harness the interactive strategies including peer and instructor supported collaboration and discussion. However, the internet services required to enable this type of interactivity may not be universally available, particularly in rural communities with poor technological infrastructure and limitations in broadband and mobile internet coverage [31]. Therefore, at present, non-networked computer-based approaches may continue to play an important role in dementia education for those practitioners who are less digitally included.

Much of the current evidence on TEDE is from practice-based settings using study designs involving pre- and post-tests [28]. Experimental methods in educational research are, however, diverse [32] and there is potential for more to be known about the role of quantitative methods that evaluate TEDE programmes. For instance, studies involving comparator groups may provide insight into the relative value of different training approaches. Furthermore, studies from higher educational settings may provide valuable sources of additional information including innovative pedagogical practices. The complexities of a review of TEDE for HSCPs will benefit by incorporating a robust framework for data synthesis and presentation of the review findings. Previous reviews of dementia education have applied Kirkpatrick’s four-level model [24, 25, 27]; a widely cited framework for evaluating educational and training interventions. Each level denotes a particular value added from training investment including learners’ *reactions* to the training; *learning gains* as knowledge, skills, and attitudinal change; practice-based *behaviour changes* following training; and the wider *results* due to the training [33].

### Aim

The aim of this review was to systematically appraise and synthesise the current experimental evidence evaluating technology-enabled dementia education programmes for health and social care practitioners. The research questions were:

- What are the experimental research methods that evaluate technology-enabled dementia education programmes for health and social care practitioners?
- What are the methodological strengths and limitations of experimental studies that evaluate technology-enabled dementia education programmes for health and social care practitioners?
- Are online and non-networked computer-based dementia education programmes beneficial across the outcomes in Kirkpatrick’s model?
- What are the delivery methods, instructional strategies, and modes of information delivery in technology-enabled dementia education programmes for health and social care practitioners?
- What instructional strategies support interactivity (communication and collaboration) in technology-enabled dementia education for health and social care practitioners?

## Methods

The design and methodology of this systematic review were informed using the Preferred Reporting Items for Systematic Review and Meta-Analysis (PRISMA) guidelines and checklist [34]. The checklist is included (Additional file 1).

### Protocol

A protocol was registered on PROSPERO (CRD42018115378) and published to describe a systematic mixed methods review detailing the characteristics *and* effectiveness of TEDE [35]. The review has been completed and submitted for publication. The current review expands on the quantitative evidence and methods to evaluate the effectiveness of TEDE for HSCPs.

### Criteria for considering studies for this review

#### Eligibility criteria

We included studies involving HSCPs with, without, or working toward a professional qualification or registration participating in TEDE in a workplace or educational setting. We did not include studies of TEDE for informal/family caregivers or people living with dementia. We included all TEDE courses, modules, and standalone resources delivered in online, computer-based, and blended learning programmes. Decision support, DVD/video, and telephonic interventions were not included. We included experimental and observational study designs that involved the systematic collection of data and comparison of intervention effects in the evaluation of TEDE programmes. This included study designs that involved one group or more than one group of participants. Study designs involving one group of participants included before-after designs; interrupted time-series designs; and repeated measure designs. Study designs involving more than one group of participants included randomised trials and non-randomised studies—including controlled before-after and time-series designs [36]. These eligible study designs were categorised into two groups: experimental and quasi-experimental studies. Studies were judged to be experimental where the investigator *randomly* allocated participants to a treatment (TEDE) and a control/comparator group. Studies were considered to be quasi-experiments where they lacked the key feature of experimental studies—randomisation. Quasi-experimental studies included those with a control/comparator group (e.g., non-randomised studies) and those without a control/comparator group (e.g., before-after studies) [37]. Non-randomised studies also included quasi-randomised studies where methods of allocation were known but were not strictly random. Quasi-experimental studies shared with experimental studies a similar purpose—to test causal hypotheses. In experimental studies, random allocation creates two or more groups that are probabilistically similar to one another; therefore, any difference between the groups is likely to be due to the ‘treatment’. This feature of experimental research is highly valued and randomised trials are considered to be the ‘gold standard’ in research [38]. Quasi-experimental studies can also aid in understanding causal effects; however, the reliability of causal claims and estimates varies across these designs and depends on how close the study conditions are to an experiment [39]. Therefore, we considered the eligible study designs in a relative hierarchy, in terms of establishing causality, and provided definitions with relevance to the review context (Table 2). We included the experimental and quasi-experimental evidence from quantitative and mixed method evaluations of TEDE. We did not include qualitative studies or studies that only evaluated TEDE programmes using descriptive narrative or survey data of participants’ general impressions. Studies published before 2005 were excluded to reflect the pace of digital change and technological progress since Web 2.0 [42] . Studies not published in the English language were excluded as resources for translation were not available.

**Table 2:** Experimental and quasi-experimental study designs

**Classification**	**Category**	**Study design**	**Definition**
Experimental	1	Randomised trials	An experimental study in which individuals or groups are allocated to different interventions using methods that are random.
Quasi-experimental	2	Interrupted time-series designs	A study that uses observation at multiple (at least three) time points before and after an intervention.
	3	Controlled before-after studies	A study in which observations are made before and after the implementation of an intervention, both in a group that receives the intervention and in a control group that does not.
	4	Non-randomised studies	A study in which people are allocated to different interventions using methods that are not random.
	5	Before-after studies	A study in which observations are made before and after the implementation of an intervention in the same group of individuals.
	5	Repeated measure studies	A before-after study in which there are multiple post-intervention time points at which outcome measurements are made [40].

Category 1 studies are considered to be the most robust, and category 5 studies the least robust, in terms of establishing causality. Hierarchies of quasi-experimental studies informed by Harris et al. [41]. Definitions of study designs were adapted from Cochrane Effective Practice and Organisation of Care (EPOC) guidance [36] unless otherwise indicated

### Outcome measures

The primary outcome measures were based on Kirkpatrick’s four-level model which is the most renowned and widely used evaluation model for educational and training interventions [43, 44]. The model was adapted for the review context to identify relevant outcomes from primary studies and to provide a framework for data synthesis and presentation of the review findings [45]. We adapted the model for greater emphasis and delineation of the level 2 sub-items (knowledge, skills, and attitudes) as each, in isolation, was considered to be an important learning outcome in the dementia education context. Definitions for each level in the adapted model are provided in Table 3.

**Table 3:** Definitions for the levels of outcome evaluation

**Level**	**Outcome**	**Definition**
1	Reaction	This is a measure of how participants feel about aspects of the TEDE programme. It is a measure of learner satisfaction.
2a	Learning-change in knowledge	This is a measure of knowledge acquired as a result of the TEDE programme.
2b	Learning-change in skills	This is a measure of the skills acquired as a result of the TEDE programme.
2c	Learning-change in attitudes	This is a measure of attitudinal change as the result of the TEDE programme.
3	Behaviours	This is a measure of the extent to which participants change their behaviours in practice because of the TEDE programme.
4	Results	This is a measure of results that occurred because of the TEDE programme. It includes outcomes for service users and other organisational-level outcomes.

*TEDE* Technology-enabled dementia education

### Search methods

Literature searches were carried out in MEDLINE (OVID interface), CINAHL Complete (EBSCO interface), ERIC (EBSCO interface), PsycINFO (EBSCO interface), PubMed, Web of Science Core Collection, OVID Nursing Database, and SCOPUS from January 2005 until November 2018. The search was updated in February 2020. MEDLINE and PubMed were both included to ensure that the search was comprehensive. MEDLINE is a subset of PubMed with the latter containing more material. Subject librarians (RP and COM) from the University of the Highlands and Islands were consulted in the development of the search strategy. Experts in TEDE were not contacted for other sources of information. The multi-database search strings are available (Additional file 2).

### Data collection and analysis

#### Selection of studies

The results from the literature search were stored in RefWorks research management software where duplicate citations were identified, confirmed, and removed. One reviewer (KM) then screened the titles and abstracts of the remaining studies. Two other reviewers (LM and CC) then screened 10% of the titles and abstracts—by each screening 5%. Eligibility conflicts were resolved through discussion and third-party arbitration was not required. Full-text versions of potentially eligible studies were assessed for eligibility by one reviewer (KM). Ineligible studies were issued with an exclusion rationale and removed. Reference lists of eligible studies were screened by KM and any studies meeting the eligibility criteria were included. One reviewer (KM) examined the eligible study reports (papers) to establish any instances where more than one paper reported on the same training programme. Papers reporting duplicate evaluations were not eligible; however, any papers that reported on different aspects (i.e., multiple evaluations) of the same training programme were eligible for inclusion.

#### Data extraction

Data was extracted using a data extraction form designed specifically for the review. The form was pilot tested before application and study data was extracted by one reviewer (KM). A sample data extraction form is provided (Additional file 3).

### Assessment of methodological quality

The Mixed Methods Appraisal Tool (MMAT) [46] and Medical Education Research Study Quality Instrument (MERSQI) [47] were used in a two-stage process for quality appraisal. MMAT is a generic critical appraisal tool with specific categories for qualitative research, randomised trials, non-randomised studies, quantitative descriptive studies, and mixed methods studies. The provision of multiple methodological quality criteria for different study designs makes it most relevant for quality appraisal in mixed studies reviews [48]. MMAT was considered to be appropriate for use in this *non-mixed studies review* as the tool was being used simultaneously by the study authors in a mixed methods review of TEDE. This was a pragmatic decision to achieve consistency between the two reviews given that MMAT has sufficient capacity to appraise the experimental design methodologies included in the current review. Each MMAT category has a specific criteria with three response options: ‘yes’ means that the criterion is met, ‘no’ means that the criterion is not met, and ‘can't tell’ means that there is not enough information in the paper to judge if the criterion is met. MERSQI puts emphasis on methodological rigour associated with experimental and quasi-experimental studies in medical education [49]. It is a reliable tool for appraising the quality of medical education research and has been used previously to complement other tools [50]. MERSQI has 6 domains: study design, sampling, type of data, validity of evaluation instrument, data analysis, and outcomes. Each domain includes items which are scored based on the methodological strengths of primary studies. The maximum domain score is 3 and the maximum total MERSQI score is 18. MMAT was used to assess the quality of individual studies and MERSQI established the overall quality of TEDE research. The combined approach to quality appraisal also aimed to highlight unique methodological features of educational research for consideration in the context of generic appraisal. Two reviewers (KM and LM or KS) appraised 19% of studies using MMAT. Disagreements were resolved through discussion. All other quality appraisal was conducted by one reviewer (KM).

### Data synthesis

All of the studies included in the review reported findings quantitatively; however, it was not appropriate to undertake meta-analyses due to the heterogeneity of study designs and statistical data. Instead, findings from the primary studies were summarised in a narrative synthesis using textual description and tabulation. A systematic approach was applied to the synthesis. Firstly, studies of online dementia education (ODE) and non-networked computer-based dementia education (CBDE) were identified and synthesised separately. For each e-learning approach, pedagogical strategies were discussed and key study information including intervention characteristics were presented in a summary table. Kirkpatrick’s model provided a framework for subsequent synthesis where shared outcomes between studies were grouped together. Study designs and outcome measures within studies were then discussed before further delineation of findings according to the study setting (i.e., practice or higher education). Study findings were discussed with a particular focus on those studies that involved comparator/control groups—allowing inference to optimal training approaches. Finally, key study findings were tabulated. The effects of interventions were reported at the level of each individual study. Each study was classified to show if the TEDE programme resulted in evidence of an effect, no evidence of an effect, or partial evidence of an effect (where a study reported inconsistent effects due to multiple outcome measures) for the outcomes measured. Effects were based on statistically significant pre- to post-test increases or between group differences that favoured treatment (TEDE) groups. Analysis of sub-groups was not undertaken; however, studies that included follow-up data were identified for inference on the sustainability of training effects over time.

## Results

### Description of studies

#### Results of the search

**Fig. 2:**
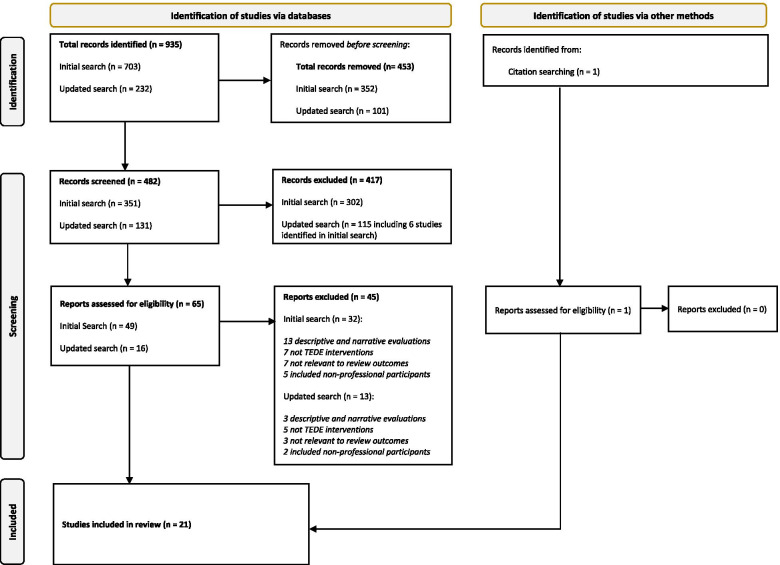
PRISMA diagram [51]

A total of 935 records were identified. Duplicate records (453) were removed and the titles and abstracts of 482 remaining records were screened based on the eligibility criteria. From these, 417 records were considered to be ineligible and the full texts of 65 records were retained for full-text review. Forty-five records were excluded as they focused on descriptive research using narrative or survey-based evaluations of TEDE programmes; did not include TEDE programmes; were not relevant to the review outcomes; or included non-HSCPs (family carers). The remaining 20 studies were included in the final synthesis with an additional study identified from the reference lists of eligible studies (Fig. 2).

### Included studies

**Fig. 3:**
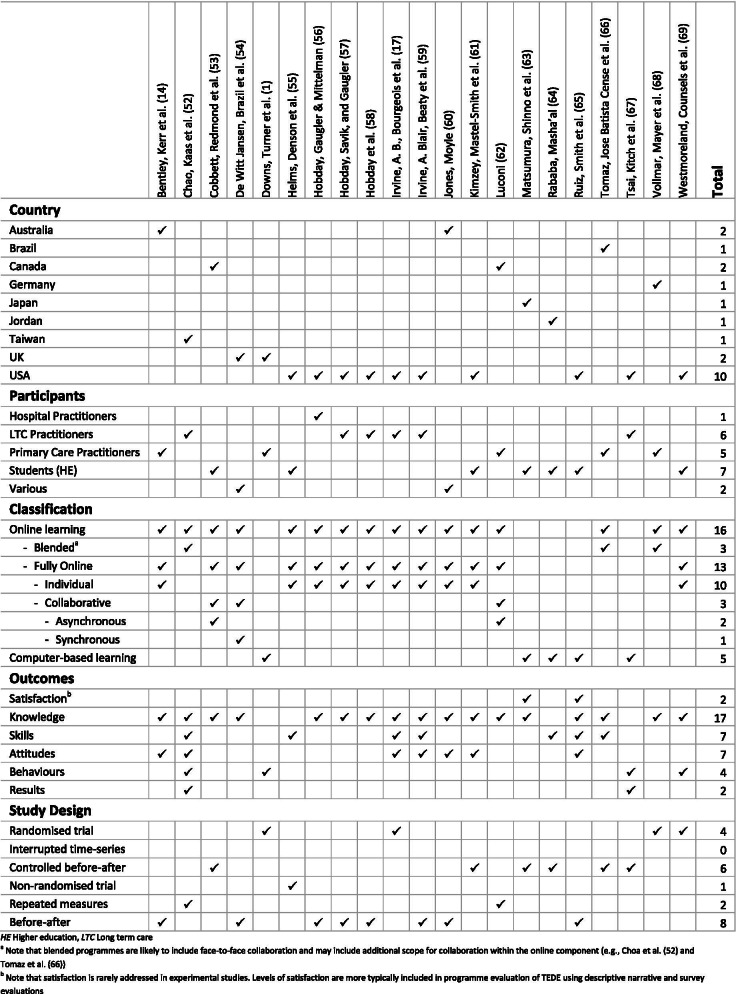
Study and intervention characteristics

Key study and intervention characteristics from 21 eligible studies are demonstrated in Fig. 3. Full details of the included studies are provided in the Characteristics of Included Studies (Additional file 4).

### Excluded studies

Studies considered ineligible for inclusion following full-text review are available. Exclusion rationales are provided (Additional file 5).

### Study designs

**Fig. 4:**
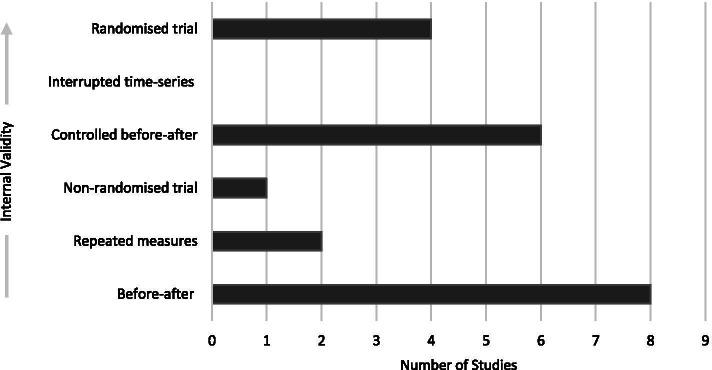
Study designs

Four studies were randomised trials [1, 17, 68, 69] of which two were cluster randomised trials [1, 68]. There were no interrupted time-series designs. Six studies were classified as being controlled before-after designs [53, 61, 63, 64, 66, 67]. Two of these studies described random allocation processes [64, 67]; however, they were not classified as randomised trials. One of the studies described random assignment to groups from a *convenience sample* [64] and the other introduced non-randomised participants into the experimental group [67]. There was one non-randomised study [55] and two studies used repeated measures [52, 62]. Eight before-after studies were included [14, 54, 56–60, 65] (Fig. 4). Six of the included studies were exploratory pilot studies [57–59, 62, 63, 67].

### Quality assessment of included studies

#### MMAT

##### Experimental studies (MMAT2)

Randomisation processes were not described sufficiently in two of the trials [17, 69]. Between group incomparability was identified in one trial [1], and it was not clear if between group similarities were significant in another [68]. An arbitrary threshold was applied for the assessment of outcome data. Acceptable dropout rates were considered to be < 20%, which negatively affected judgments on the quality of three of the trials [1, 68, 69]. One trial was unblinded [1], and it was not possible to tell if outcome assessors were blinded in two trials [68, 69]. Participant adherence may have been compromised in an unsupervised online dementia training [17]. Non-adherence was more apparent where intervention ‘non-users’ were identified [68], and where a trial ended prematurely due to participant dissatisfaction [69].

##### Quasi-experimental studies (MMAT3)

There were a number of reporting limitations in quasi-experimental studies. It was frequently not possible to determine if participants were representative of target populations. Sampling methods were often not described, or there was insufficient information in relation to sampling or target populations. Convenience sampling was particularly problematic when assessing representativeness. It was frequently not possible to determine if outcome measures were appropriate. Limitations included inadequate reports of validity or reliability (e.g., [55]); partial reporting of valid/reliable measures in studies using multiple measures (e.g., [60]); reports of validated measures that may not be reliable and vice versa (e.g., [56]); previously validated measures that were not validated in context (e.g., [14]); and measures with questionable reliability from sub-optimal alpha levels (e.g., [53]). An arbitrary threshold was applied to determine the completeness of outcome data. Acceptable dropout rates were considered to be < 20%. It was frequently not possible to tell if the outcome data was complete. This was a common issue in before-after studies due to suboptimal reporting of participant numbers at either pre- or post-test. Two studies reported outcome data below the desired threshold [57, 67]. Interventions were considered to have been administered as intended unless studies reported evidence to the contrary. Cannot tell judgments were generally applied to studies that reported limitations to study processes (e.g., [52]); or where there were insufficient assurances of intervention controls including the location of participation (e.g., [55]). The main threat to study quality was from confounding factors which were frequently not described or accounted for in the study design or analysis. Time difference between pre- and post-tests in before-after studies was a common source of potential maturation effects.

**Fig. 5:**
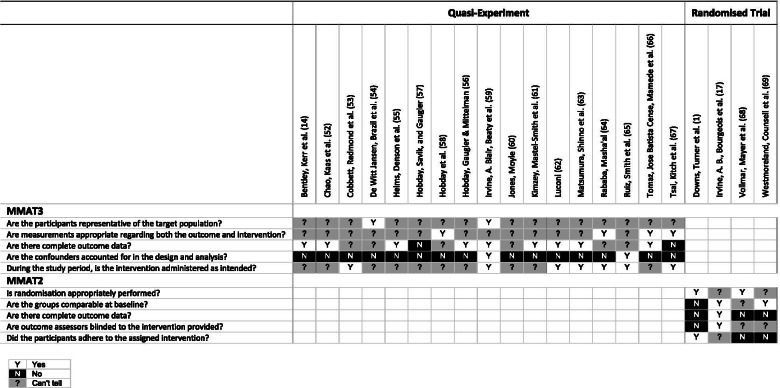
Quality appraisal with Mixed Methods Appraisal Tool

A summary of MMAT quality appraisal is shown in Fig. 5. The ratings and support for judgment are provided in the Characteristics of Included Studies (Additional file 4).

#### MERSQI

The mean MERSQI scores from primary studies are presented with the standard deviation (SD). Scoring information is available for each domain and subdomain. The total MERSQI score was 12.38 (SD 1.6) which was interpreted to be ‘moderate’ quality overall (Table 4). Scoring data for individual primary studies and a decision-making tool developed for consistency across the review context are provided (Additional file 6).

**Table 4:** Medical Education Research Study Quality Instrument: domain and total scores

	**Possible score**	**Mean (SD)**
**Study design**	**3**	1.95 (0.6)
Single group cross‐sectional or single group post-test only	1	
Single group pre-test and post-test	1.5	
Nonrandomised, 2 groups	2	
Randomised controlled trial	3	
**Sampling**		
**Institutions studied**	**1.5**	1.05 (0.5)
1	0.5	
2	1	
3	1.5	
**Response rate, %**	**1.5**	1.31 (0.4)
Not applicable	0	
< 50 or not reported	0.5	
50–74	1	
> 75	1.5	
**Type of data**	**3**	2.33 (0.9)
Assessment by participants	1	
Objective measurement	3	
**Validity of evaluation instrument**		
**Internal structure**	**1**	0.52 (0.5)
Not applicable	0	
Not reported	0	
Reported	1	
**Content**	**1**	0.52 (0.5)
Not applicable	0	
Not reported	0	
Reported	1	
**Relationships to other variables**	**1**	0.05 (0.2)
Not applicable	0	
Not reported	0	
Reported	1	
**Data analysis**		
**Appropriateness of analysis**	**1**	1.00 (0.0)
Inappropriate for study design or type of data	0	
Appropriate for study design and type of data	1	
**Complexity of analysis**	**2**	1.95 (0.2)
Descriptive analysis only	1	
Beyond descriptive analysis	2	
**Outcomes**	**3**	1.69 (0.4)
Satisfaction, attitudes, perceptions, opinions, general facts	1	
Knowledge, skills	1.5	
Behaviours	2	
Patient/healthcare outcome	3	
**Total**	**18**	**12.38 (1.6)**

*SD* Standard deviation

### Establishing the effectiveness of TEDE

From 21 studies identified in this review, 16 studies described ODE programmes [14, 17, 52–62, 66, 68, 69] and 5 involved non-networked CBDE approaches [1, 63–65, 67].

### Online dementia education programmes

Twelve studies of ODE were from practice-based settings [14, 17, 52, 54, 56–60, 62, 66, 68] and 4 were from higher education [53, 55, 61, 69]. Thirteen studies reported on fully online programmes [14, 17, 53–62, 69] and 3 included blended learning approaches [52, 66, 68]. Thereafter, inconsistent use of terminology (e.g., resources, programmes, courses, or modules) between studies made classification approaches more challenging. In general, online courses were either labelled as such [53], or involved a protracted duration [62, 66]. Modules were generally described in terms of duration and frequency [14, 54, 57–60, 68]. Frequently, duration and frequency were not specified [17, 52, 55, 56, 61, 69].

#### Interactivity

Two studies of blended learning stipulated additional scope for interactivity within the online components [52, 66]. One specified discussions in asynchronous virtual forums and synchronous chat facilities [66]. The other described online peer discussions including internet-based 360° feedback and reflective journaling among participants and the programme instructor [52]. Interactions in fully online approaches were predominantly from moderator facilitated asynchronous discussion boards [53, 62]. One programme allowed for synchronous discussion by virtue of being an interactive videoconferencing course [54].

#### Instructional strategies

Case-based instruction was the most frequently described instructional strategy regardless of educational setting or delivery method [17, 52–54, 56, 59, 60, 62, 66, 68, 69]. Practice-based and problem-based learning were described in two studies involving family physicians [62, 66]. Luconi [62] described the practice-based learning method evolving from problem-based learning. These approaches were applied to fully online [62] and blended approaches [66]. They were not described within the higher education context.

#### Mode of information delivery

Video was the most frequently described mode of information delivery [14, 17, 52, 53, 55–59, 66, 69]. This was a common approach in all educational settings. Video was used to present scenarios [52, 56] and to present discussions with HSCPs, people living with dementia, and dementia experts [14, 56]. Unscripted, real-life videos involving people living with dementia, their families, and carers were highlighted [56, 57]. Video was used to demonstrate skills [69] including video-modelling techniques to hone dementia care skills among practitioners [17, 59]. Video also provided a mode of delivery for more traditional lectures [66]. Lectures were also described within the face-to-face component of a blended learning programme [52] and were a feature of an online videoconferencing programme [54].

Textual delivery of information was described in practice-based programmes [17, 56–59, 62] and in higher educational contexts [53, 55, 69]. In higher education, textual information was described as being explanatory [55]. Practice-based programmes were more likely to address staff literacy by including audio-narrated text [56, 58], basing text on 2nd to 8th grade reading levels [17, 56, 59], or by using short titles and bullet points [59]. Narration [17, 59], audio [57], and graphics [17, 56, 58, 69] were other modes of information delivery described. Few studies provided links to additional or external learning resources [14, 55, 62].

#### Assessment

Assessment strategies were mostly described within practice-based ODE programmes. These included questions [14, 52], multiple choice questions (MCQ) [17, 62], interactive exercises [58], and interactive text entry [56]. Quizzes, more generally, were described in programmes from both practice-based and higher educational settings [53, 55, 62].

The key programmes characteristics in ODE are summarised in Table 5. Programme characteristics are unlikely to be fully representative due to underreporting in the primary studies.

**Table 5:** Key characteristics of online dementia education programmes

**Study**	**Setting/participants**	**Programme**	**Duration**	**Programme characteristics**
Bentley, Kerr et al. 2019 [14]	Primary care International medical graduates and practice nurses	Online interactive educational resource (recognising, diagnosing, and managing dementia in general practice)	4 modules Total duration: 3 h	**Delivery method:** fully online **Instructional strategy:** NS **MID:** video; external learning resources **Interactivity:** NS
Chao, Kaas et al. 2016 [52]	Long-term care Nurses	Advanced Innovative Internet-Based Communication Education Program	4 modules Total duration NS	**Delivery method:** blended learning **Instructional strategy:** case-based **MID:** video; face-to-face **Interactivity:** face-to-face discussion; online discussion NOS; online reflective journaling
Cobbett, Redmond et al. 2016 [53]	Higher education Nursing students	Alzheimer’s disease and other dementias care course: adapted online course	8 online modules and 1 face-to-face^a^ Module duration: 1 h (with additional 2 h of preparatory work)	**Delivery method:** fully online^a^ **Instructional strategy:** case-based **MID:** video; text **Interactivity:** online discussion (asynchronous)
De Witt Jansen, Brazil et al. 2018 [54]	Primary and Secondary care, Nursing Home, and Hospice Physicians and nurses	Tele-mentoring to enhance assessment and management of pain in advanced dementia (based on Project ECHO model)	5 sessions Session duration: 1 h 15 min	**Delivery method:** fully online **Instructional strategy:** case-based **MID:** videoconferencing **Interactivity:** online discussion (synchronous)
Helms, Denson et al. 2009 [55]	Higher education Medical students	E-module: Neurology and Dementia: Psychological Aspects of Care (with clerkship materials)	Total duration NS	**Delivery method:** fully online^b^ **Instructional strategy:** NS **MID:** video; text; external learning resources **Interactivity:** NS
Hobday, Savik, and Gaugler 2010 [57]	Long-term care Direct care workers	Internet-based multimedia education program: dementia training resource	3 prototype modules Module duration: 1 h	**Delivery method:** Fully Online **Instructional strategy:** NS **MID:** video; text; audio **Interactivity:** NS
Hobday et al. 2010 [58]	Long-term care Nurse assistants	Internet-based, interactive, multimedia dementia educational program	4 modules Module duration: 1 h	**Delivery method:** fully online **Instructional strategy:** NS **MID:** video; text; audio-narration; graphics **Interactivity:** NS
Hobday, Gaugler and Mittelman 2017 [56]	Hospital Nursing assistants and allied hospital workers	CARES® dementia-friendly hospital program: online dementia training program	4 modules Total duration NS	**Delivery method:** fully online **Instructional strategy:** case-based **MID:** video; audio-narrated text; text considers literacy levels; graphics **Interactivity:** NS
Irvine, A. B., Bourgeois et al. 2007 [17]	Long-term care Nurse aides	Interactive multimedia program: professional dementia care (managing aggression)	Total duration NS	**Delivery method:** fully online **Instructional strategy:** video-modelling **MID:** video; text considers literacy levels; narration; graphics **Interactivity:** NS
Irvine, A. Blair, Beaty et al. 2013 [59]	Long-term care Non-direct care staff including nurses	Internet dementia-training program	5 modules 2 h to complete all modules	**Delivery method:** fully online **Instructional strategy:** video-modelling **MID:** video; text considers literacy levels; narration **Interactivity:** NS
Jones, Moyle 2016 [60]	Long-term care Nurses, care workers, and students	Online self-directed e-learning education intervention (based on the sexualities and dementia education resource for health professionals)	4 modules Module duration: 1 h	**Delivery method:** fully online **Instructional strategy:** case-based **MID:** NS **Interactivity:** NS
Kimzey, Mastel-Smith et al. 2016 [61]	Higher education Nursing students	Alzheimer’s disease online module	Total duration NS	**Delivery method:** fully online **Instructional strategy:** NS **MID:** NS **Interactivity:** NS
Luconi 2008 [62]	Primary care Family physicians	Early Alzheimer’s disease program: web-based continuing medical education program	8 modules completed over 6 months	**Delivery method:** fully online **Instructional strategy:** case-based; practice-based learning method **MID:** text; external learning resources **Interactivity:** online discussions (asynchronous); moderated
Tomaz, Jose Batista Cisne, Mamede et al. 2015 [66]	Primary care Family physicians	Online PBL: clinical approach for elderly with dementia	120 h (100 h distance and 20 face-to-face) over 12 weeks	**Delivery method:** blended learning^c^ **Instructional strategy:** case-based; PBL **MID:** video; face-to-face **Interactivity:** face-to-face discussion; online discussions (synchronous; asynchronous)
Vollmar, Mayer et al. 2010 [68]	Primary care General practitioners	Online modules: presentation of a dementia guideline	Estimated average activity duration: 83 (15 to 200) min	**Delivery method:** blended learning **Instructional strategy:** case-based **MID:** online NOS **Interactivity:** face-to-face discussions
Westmoreland, Counsell et al. 2010 [69]	Higher education Medical residents	Dementia education module within a web-based geriatrics training program	Total duration NS	**Delivery method:** fully online **Instructional strategy:** case-based **MID:** video; text; graphics **Interactivity:** NS

*MID* Mode of information delivery, *NOS* Not otherwise specified, *NS* Not stated/specified, *PBL* Problem-based learning

^a^The final module was delivered through a face-to-face presentation—not otherwise classified as blended learning

^b^Multiple classifications used. Classified as fully online dementia education as authors report the efficacy of an ‘online module’ and conclude the utility of online e-modules

^c^This study included substantial face-to-face learning (20 h) and was classified as blended learning on this basis

#### Effect of ODE on learner satisfaction

None of the included studies reported on learners’ reaction to ODE programmes using experimental or quasi-experimental methods. Ten studies included reports of learner satisfaction in additional narrative or survey evaluations [14, 17, 53, 54, 56–60, 62].

#### Effect of ODE on knowledge

Fifteen studies evaluated the effects of ODE programmes on learners’ knowledge [14, 17, 52–54, 56–62, 66, 68, 69]. Four studies described validated instruments for outcome measurement: the Alzheimer’s Disease Knowledge Scale [61], the Ageing Sexual Knowledge and Attitudes Scale [60], the Communication Knowledge Scale [52], and the Dementia Knowledge Assessment Scale [14]. Only one instrument, a Chinese version of the Communication Knowledge Scale, was validated for the study context [52]. Eight studies created study specific instruments for outcome measurement and provided evidence of validation [53, 54, 56–58, 62, 66, 69]. The validity of outcome measures were not reported in three studies [17, 59, 68]. Where outcome measures were reported, there was inconsistent reporting of validity and reliability. One study used more than one outcome measure [62]. Twelve studies were from practice settings [14, 17, 52, 54, 56–60, 62, 66, 68] and three were from higher education [53, 61, 69]. Practice-based studies involved practitioners from long-term care [17, 52, 57–60], primary care [14, 62, 66, 68], and hospital [56]. One study involved practitioners from a variety of healthcare settings [54]. Three practice-based studies compared outcomes between intervention and control/comparator groups [17, 66, 68]. Modest knowledge gains were reported among GPs who completed a blended learning programme consisting of online modules and structured discussions compared to those who attended a traditional lecture and structured discussions; however, the difference between the groups was not statistically significant [68]. Knowledge gains were significantly greater among long-term care practitioners who participated in an internet-based training using video-modelling and mastery learning compared to a control group who did not participate in training [17]. Family physicians demonstrated significantly improved knowledge following participation in a blended approach to problem-based learning compared to a control group who did not receive the training [66]. Nine of the practice-based studies did not involve comparator/control groups and evaluated differences in practitioners’ knowledge before and after ODE [14, 52, 54, 56–60, 62]. All of these studies demonstrated improvements in practitioner knowledge following ODE. Three studies of ODE were conducted in higher education settings involving either nursing students [53, 61] or medical residents [69]. All studies from higher education compared outcomes between intervention and control/comparator groups. Statistically significant knowledge gains were established among medical residents where a web-based dementia education module was compared to paper-based learning [69]. Knowledge gains were significantly greater among nursing students who completed an ODE programme compared to a control group who did not [53]. In the third study, nursing students demonstrated modest post-test knowledge gains following an Alzheimer’s Disease online module whereas the control group, who did not receive any form of dementia training, did not. It is of note that this study involved an additional experiential arm of students who completed learning in practice. The experiential group demonstrated greater knowledge gains compared to the ODE group. Only the findings for the experiential group were statistically significant [61]. The key study characteristics and findings for the knowledge-based outcomes following ODE are summarised in Table 6.

**Table 6:** Study characteristics and findings for knowledge-based outcomes following online dementia education

**Study**	**Setting**	**Study design**	**Comparator**	**Measure**	**Results**
Bentley, Kerr et al. 2019 [14]	Practice	Before-after	NA	DKAS	Evidence of effect^a^
Chao, Kaas et al. 2016 [52]	Practice	Repeated measures	NA	CKS-C	Evidence of effect^b^
Cobbett, Redmond et al. 2016 [53]	HE	Controlled before-after	Did not participate in online training	MCQ^c^	Evidence of effect
De Witt Jansen, Brazil et al. 2018 [54]	Practice	Before-after	NA	ECHO Questionnaire^d^	Evidence of effect
Hobday, Savik, and Gaugler 2010 [57]	Practice	Before-after	NA	Knowledge Inventory	Evidence of effect
Hobday et al. 2010 [58]	Practice	Before-after	NA	Dementia Care Knowledge	Evidence of effect
Hobday, Gaugler and Mittelman 2017 [56]	Practice	Before-after	NA	Dementia Care Knowledge	Evidence of effect
Irvine, A. B., Bourgeois et al. 2007 [17]	Practice	Randomised trial	Did not participate in training	VST: knowledge	Evidence of effect
Irvine, A. Blair, Beaty et al. 2013 [59]	Practice	Before-after	NA	VST: knowledge	Evidence of effect^e^
Jones, Moyle 2016 [60]	Practice	Before-after	NA	ASKAS: knowledge	Evidence of effect
Kimzey, Mastel-Smith et al. 2016 [61]	HE	Controlled before-after	Usual practice; Experiential learning	ADKS	No evidence of effect
Luconi 2008 [62]	Practice	Repeated measures	NA	MCQ; clinical cases	Partial evidence of effect^b^ (*multiple outcome measures*)
Tomaz, Jose Batista Cisne, Mamede et al. 2015 [66]	Practice	Controlled before-after	Did not receive training	Knowledge test	Evidence of effect
Vollmar, Mayer et al. 2010 [68]	Practice	Randomised trial	Traditional lecture	Knowledge test	No evidence of effect
Westmoreland, Counsell et al. 2010 [69]	HE	Randomised trial	Paper-based learning	Knowledge test	Evidence of effect

*ADKS* Alzheimer’s Disease Knowledge Scale, *ASKAS* Ageing Sexual Knowledge and Attitudes Scale, *CKS-C* Communication Knowledge Scale–Chinese version, *DKAS* Dementia Knowledge Assessment Scale, *HE* Higher education, *MCQ* Multiple choice question, *NA* Not applicable, *VST* Video situation test

^a^Based on descriptive statistics

^b^Based on first time point following training

^c^Measured comprehension, application, and critical thinking

^d^Measured knowledge and self-efficacy

^e^The findings related to nurses only

#### Effect of ODE on skills

Five studies evaluated the impact of ODE on learners’ skills [17, 52, 55, 59, 66]. Outcomes were assessed using a variety of measures. One study reported reliable measures to assess the application of the mini-mental state exam (MMSE) and skills in differential diagnosis [66]. In another study, the research team developed and validated the Communication Competency Scale [52]. The remaining studies did not provide evidence of validated outcome measures [17, 55] although one study reported alpha statistics providing some evidence of reliability [59]. Four studies used more than one outcome measure [17, 55, 59, 66]. Four studies were conducted in the practice setting [17, 52, 59, 66]. The practice-based studies involved practitioners from long-term care [17, 52, 59] and primary care [66]. Two practice-based studies compared outcomes between intervention and control/comparator groups [17, 66]. Family physicians who participated in the blended approach to problem-based learning demonstrated significantly improved skills in differential diagnosis and mini-mental state examinations when compared to a control group who did not receive the training [66]. Nurse aides who completed the internet-based training using video-modelling demonstrated improved self-efficacy regarding distressed resident behaviours when compared to a control group who did not receive the training [17]. We classified self-efficacy as a ‘skill’ as it is often concerned with judgments of how well one can *execute a course of action* required to deal with situations [70]; furthermore, it has received similar classification in the existing literature on TEDE [65]. The other two practice-based studies did not involve comparator/control groups and evaluated differences in practitioners’ skills before and after ODE [52, 59]; however, only one study demonstrated improvements in practitioners’ skills [52]. One study was from higher education and involved medical students who completed an e-module on psychosocial aspects of dementia care and subsequently performed better in an OSCE when compared to a control group who did not participate in the training [55]. The key study characteristics and findings for the skills-based outcomes following ODE are summarised in Table 7.

**Table 7:** Study characteristics and findings for skills-based outcomes following online dementia education

**Study**	**Setting**	**Study design**	**Comparator**	**Measure**	**Results**
Chao, Kaas et al. 2016 [52]	Practice	Repeated measures	NA	CCS	Evidence of effect^a^
Helms, Denson et al. 2009 [55]	HE	Non-randomised trial	Did not participate in e-module	OSCE clinical note score; OSCE performance	Evidence of effect
Irvine, A. B., Bourgeois et al. 2007 [17]	Practice	Randomised trial	Did not participate in training	Self-efficacy; VST: Self efficacy	Evidence of effect
Irvine, A. Blair, Beaty et al. 2013 [59]	Practice	Before-after	NA	Self-efficacy; VST: Self efficacy	No evidence of effect^b^
Tomaz, Jose Batista Cisne, Mamede et al. 2015 [66]	Practice	Controlled before-after	Did not receive training	DD; MMSE	Evidence of effect

*CCS* Communication Competency Scale, *DD* Differential diagnosis, *HE* Higher education, *MMSE* Mini-mental state examination, *NA* Not applicable, *OSCE* Objective structured clinical examination, *VST* Video situation tests

^a^Based on first time point following training

^b^The findings relate to nurses only

#### Effect of ODE on attitudes

Six studies evaluated the impact of ODE on learners’ attitudes [14, 17, 52, 59–61]. Four studies used previously validated instruments for outcome measures: the Confidence and Attitudes Towards Dementia Scale [14], the Communications Skills Attitudes Scale [52], the Aging Sexual Knowledge and Attitude Scale [60], and the Dementia Attitudes Scale [61]. The Communications Skills Attitudes Scale–Chinese version was validated for the study context [52]. One study used the Staff Attitudes about Intimacy and Dementia Survey although the psychometric properties were undetermined [60]. The remaining studies used programme-specific outcome measures; one was unvalidated [17] and one provided evidence of reliability only [59]. Three studies used more than one outcome measure [17, 59, 60]. Five studies were conducted in the practice setting [14, 17, 52, 59, 60]. The practice-based studies involved practitioners from long-term care [17, 52, 59, 60] and primary care [14]. Only one of the practice-based studies compared outcomes between intervention and control/comparator groups.

The nurse aides who completed internet-based training using video-modelling demonstrated improved attitudes and behavioural intentions regarding distressed resident behaviours when compared to a control group who did not receive the training [17]. Behavioural interventions were classified as ‘attitudes’ as, according to the Theory of Reasoned Action, attitudes are postulated as direct determinants of behavioural intentions [71]. Four practice-based studies did not involve comparator/control groups and evaluated differences in practitioners’ attitudes before and after ODE [14, 52, 59, 60]. Improved staff attitudes were observed following the training in all but one study [52].

One study was conducted in higher education and compared attitudinal change among nursing students who either completed an ODE programme or received no dementia specific intervention. Modest and non-significant attitudinal change was observed in both groups. It is of note that this study involved an additional arm comprising of students who completed experiential learning in practice. The experiential group demonstrated statistically significant improvements in attitudes toward people with dementia [61]. The key study characteristics and findings for the studies reporting attitudinal change following ODE are summarised in Table 8.

**Table 8:** Study characteristics and findings for studies reporting attitudinal change following online dementia education

**Study**	**Setting**	**Study design**	**Comparator**	**Measure**	**Results**
Bentley, Kerr et al. 2019 [14]	Practice	Before-after	NA	GPACS-D	Evidence of effect^a^
Chao, Kaas et al. 2016 [52]	Practice	Repeated measures	NA	CSAS-C	No evidence of effect^b^
Irvine, A. B., Bourgeois et al. 2007 [17]	Practice	Randomised trial	Did not participate in training	Attitudes; behavioural Intentions	Evidence of effect
Irvine, A. Blair, Beaty et al. 2013 [59]	Practice	Before-after	NA	Attitudes; behavioural intentions	Partial evidence of effect^c^ (*multiple outcome measures*)
Jones, Moyle 2016 [60]	Practice	Before-after	NA	ASKAS: attitude; SAID	Evidence of effect
Kimzey, Mastel-Smith et al. 2016 [61]	HE	Controlled before-after	Usual practice; experiential learning	DAS	No evidence of effect

*ASKAS* Ageing Sexual Knowledge and Attitudes Scale, *CSAS-C* Communications Skills Attitudes Scale–Chinese version, *DAS* Dementia Attitudes Scale, *GPACS-D* Confidence and Attitudes Towards Dementia Scale, *HE* Higher education, *NA* not applicable, *SAID* Staff Attitudes about Intimacy and Dementia Survey

^a^Based on descriptive statistics

^b^Based on first time point following training

^c^The findings related to nurses only

#### Effect of ODE on behaviours

Two studies evaluated the impact of ODE on learners’ behaviours [52, 69]. One study used multiple outcome measures and did not report on the validation of the measures used [69]. The other study reported a reliable and valid outcome measure [52]. The studies were carried out in long-term care [52] and higher education [69]. In long-term care, the frequency in which nurses assessed their patients’ communication ability was assessed before and after the blended learning programme on communication between nurses and patients with dementia. The results indicated that a higher frequency of assessments was conducted following the training [52]. In higher education, postgraduate medical residents’ behaviours were evaluated in interactions with unannounced standardised patients *in the practice setting* following the web-based dementia education module. Outcome measures included an encounter checklist, chart abstraction scores, and treatment orders placed on an electronic medical record system. Only residents’ chart abstraction scores were significantly better when compared with a comparator group that participated in paper-based learning [69]. The key study characteristics and findings for the behaviour-based outcomes following ODE are summarised in Table 9.

**Table 9:** Study characteristics and findings for behaviour-based outcomes following online dementia education

**Study**	**Setting**	**Study Design**	**Comparator**	**Measure**	**Results**
Chao, Kaas et al. 2016 [52]	Practice	Repeated measures	NA	PREAS	Evidence of effect^a^
Westmoreland, Counsell et al. 2010 [69]	HE	Randomised trial	Paper-based learning	Chart abstraction; encounter checklist; EOES	Partial evidence of effect (*multiple outcome measures*)

*EOES* Electronic Order Entry Score, *HE* Higher education, *NA* Not applicable, *PREAS* Patients Receptive and Expressive Ability Assessment Scale

^a^Based on first time point following training

#### Effect of ODE on results

The study that evaluated blended learning to improve communication between nurses and patients with dementia also included patient-level outcomes. This study evaluated differences in resident behaviours before and after practitioners received the training using two outcome measures: the Revised Memory and Behaviour Problems Checklist–Chinese version and the Cornell Scale for Depression in Dementia–Chinese version (CSDD-C). Validation of the CSDD-C was reported and both measures were reliability tested in context. The findings suggested that depressive symptoms but not behavioural problems improved at 4 weeks after the training; however, the findings were not statistically significant for either of the outcomes measured [52].

### Computer-based dementia education programmes

Two non-networked CBDE programmes were from practice-based settings [1, 67] and three were from higher education [63–65]. The CBDE programmes were either dementia training delivered on a CD-ROM [1, 65] or were computer-based simulation activities [63, 64, 67]. CD-ROM trainings were described as educational tutorials [1] or multimedia training [65]. Simulation activities included a clinic simulator involving virtual patients [63], a video simulator modelling appropriate levels of dressing assistance [67], and computer-based branching path simulation (BPS). BPS is interactive learning tool that can develop critical thinking skills and decision-making capability among learners [64]. Key characteristics of the CBDE programmes are summarised in Table 10.

**Table 10:** Key characteristics of computer-based dementia education programmes

**Study**	**Setting/participants**	**Programme**	**Duration**	**Programme characteristics**
Downs, Turner et al. 2006 [1]	Primary care GP practices	Educational tutorial on CD-ROM	Total duration: NS	**Delivery methods**: CD-ROM **Instructional strategy**: case-based **MID:** electronic book (hypertext indexing system)
Matsumura, Shinno et al. 2018 [63]	Higher education Medical students	Clinic simulator with virtual patients (alongside conventional learning)	Simulator duration: 0.75 h	**Delivery methods:** Windows 7 Operating System **Instructional strategy:** Simulated learning; case-based **MID:** 3D simulation; text; video
Rababa, Masha'al 2020 [64]	Higher education Nursing students	Computer-based BPS for pain management in people with dementia	6 sessions Session duration: 1-h duration	**Delivery methods**: computer-based **Instructional strategy**: BPS; case-based **MID**: NS
Ruiz, Smith et al. 2006 [65]	Higher education Nursing students	Multimedia training CD-ROM: Alzheimer’s and other Dementias	7 modules Module duration: 20–30 min	**Delivery methods:** CD-ROM **Instructional strategy:** NS **MID:** video; text; graphics; audio
Tsai, Kitch et al. 2018 [67]	Long-term care Nursing Assistants and Residents	Computer-based simulation—appropriate level of dressing assistance for people with dementia (with conventional learning)	Simulator duration: 2 h	**Delivery methods:** tablet device **Instructional strategy:** simulated learning; video modelling **MID:** video; text

*BPS* Branching path simulation, *MID* Mode of information delivery, *NS* Not stated/specified

#### Effect of CBDE on learner satisfaction

Learner satisfaction was included in an evaluation of the clinic simulator involving virtual patients with dementia. Medical students’ *motivation for learning* was measured using a Japanese language version of the Attention, Relevance, Confidence, and Satisfaction (ARCS) motivational model. Mean values increased significantly in all four ARCS categories after learners’ experience with the simulator [63]. In a study of CBDE using a multimedia CD-ROM, nursing students reported statistically significant improvements in pre- to post-training ratings for utility and comfort with computer-based training in general [65]. Four studies of CBDE reported on aspects of learner satisfaction in additional descriptive narrative or survey evaluations [63–65, 67].

#### Effect of CBDE on knowledge

Two studies evaluated the effects of CBDE programmes on learners’ knowledge [63, 65]. Both studies measured outcomes using a knowledge test. One of the measures was a study specific evaluation tool with evidence of validation [63]. The validity of the other measure was not reported [65]. Both studies were from higher education. One study compared the before and after scores of medical students who participated in a clinic simulator with the scores of a control group who did not participate in the simulator experience. The intervention group had significantly higher scores after the training [63]. The other study did not involve a control group and compared nursing students’ knowledge before and after a multimedia training CD-ROM [65]. A statistically significant increase in learners’ knowledge was observed following the training.

#### Effect of CBDE on skills

Two studies evaluated the impact of CBDE programmes on learners’ skills [64, 65]. One study measured outcomes using the previously validated Critical Thinking Self-Assessment Scale (CTSAS) [64]. The other used a 7-item questionnaire to measure self-reported self-efficacy in dementia care skills and did not provide evidence of validation [65]. Both studies were from higher education and involved nursing students. One study compared critical thinking skills between an intervention group with a control group before and after computer-based BPS for pain management in people with dementia. After the training, CTSAS scores in the intervention group were significantly higher than the control group [64]. The other study did not involve a control group and compared nursing students’ self-efficacy in dementia care before and after a multimedia training CD-ROM. Self-reported self-efficacy scores increased significantly after the training [65].

#### Effect of CBDE on attitudes

The study reporting on the multimedia training CD-ROM also measured attitudinal change among nursing students before and after the training. The measurement tool was a single questionnaire item designed to assess participants desire to provide care to people with dementia. Participant responses indicated an increased desire to provide care to people with dementia after the training. The findings were statistically significant [65].

#### Effect of CBDE on behaviours

Two studies assessed practitioner behaviours following CBDE [1, 67]. One study reported on the reliability of the outcome measure [67]. In the other study, practitioner behaviour was assessed using data from an electronic clinical records system [1]. Both studies were from practice settings; one from long-term care [67] and the other from primary care [1]. In long-term care, nursing assistants learned appropriate levels of dressing assistance to give their residents using a video simulator. Following the simulation activity, the nursing assistants provided more appropriate levels of assistance compared to a control group; however, the comparative difference was not statistically significant [67]. In primary care, dementia diagnosis rates among primary care practitioners were assessed following engagement with an electronic tutorial on CD-ROM, practice-based workshops, or decision support software. When compared to a control group, only the workshop and decision support interventions resulted in significant improvements in diagnosis rates. The study also reported on concordance rates with clinical guidelines for diagnosis and management of dementia. There were no significant differences between the groups studied [1].

#### Effect of CBDE on results

Tsai et al. [67] also evaluated wider results (patient-level outcomes) following the video simulator activity. Residents’ dressing performance abilities were measured using the previously validated and reliability tested Beck Dressing Performance Scale. The findings demonstrated pre- to post-training improvements in dressing performance that were greater in the intervention group compared to the control group. The difference between the groups was not statistically significant.

The key study characteristics and findings from non-networked CBDE programmes are shown in Table 11.

**Table 11:** Study characteristics and findings for all outcomes following computer-based dementia education

**Study**	**Setting**	**Study design**	**Comparator**	**Outcome**	**Measure**	**Results**
Downs, Turner et al. 2006 [1]	Practice	Randomised trial	Workshop; decision support; control (no training intervention)	Behaviours	Detection rates; concordance with guidelines	No evidence of effect
Matsumura, Shinno et al. 2018 [63]	HE	Controlled before-after	Did not participate in clinic simulator activity	Satisfaction Knowledge	ARCS Knowledge test	Evidence of effect Evidence of effect
Rababa, Masha'al 2020 [64]	HE	Controlled before-after	Traditional lectures	Skills	CTSAS	Evidence of effect
Ruiz, Smith et al. 2006 [65]	HE	Before-after	NA	Satisfaction Knowledge Skills Attitudes	PPT Knowledge test Self-efficacy test Attitude test	Evidence of effect Evidence of effect Evidence of effect Evidence of effect
Tsai, Kitch et al. 2018 [67]	Practice	Controlled before-after	Did not participate in video simulator activity	Behaviours Results	Level of dressing assistance BDPS	No evidence of effect No evidence of effect

*ARCS* Attention, Relevance, Confidence, and Satisfaction Motivational Model, *BDPS* Beck Dressing Performance Scale, *CTSAS* Critical Thinking Self-Assessment Scale, *HE* Higher education, *NA* Not applicable, *PPT* Pre- and post-training

### Sustainability of the learning outcomes

Four studies used repeated measures or included additional follow-up (FU) data which allowed for inference into the sustainability of learning outcomes following TEDE [52, 55, 62, 68]. Luconi [62] aimed to understand if family physicians’ knowledge was maintained following a web-based programme on early Alzheimer’s Disease. Pre-test and post-test mean scores from MCQ and clinical cases were compared with scores at one month post-test. Pre-test to FU MCQ scores improved significantly (pre-FU − 4.58, *p* = 0.002); however, the difference from post-test to FU was not statistically significant (post-FU − 0.43, *p* = 0.497). There were no significant differences between the pre-test or post-test to FU scores for problem-solving of clinical cases. Chao et al. [52] reported data for multiple outcomes at 16 weeks following their programme to promote communication between nurses and patients with dementia. This FU data suggested that nurses’ communication knowledge and frequency of assessing patients’ communication abilities were sustained until at least week 16. Improvements in nurses’ attitudes and communication competencies were not apparent over time. The frequency of patients’ behavioural and depressive symptoms was observed to decreased at 16 weeks compared to baseline. Vollmar et al. [68] included FU analysis of GPs’ knowledge following a blended learning programme at four months following the post-test. The mean pre-test minus FU difference was calculated to be − 2.39 in the intervention group and − 2.00 in the control group; however, the difference between groups was not significant (*p* = 0.526). Helms [55] adopted a different approach for inference of sustainability of training effects. Data from the intervention group of medical students who participated in a dementia e-module were divided into two subgroups; one subgroup took an OSCE immediately after training (immediate group) and another group who took the OSCE 1 month after training (delayed group). There was a significantly higher OSCE (performance) score in the delayed group compared to the immediate group (*p* = 0.04). The delayed group also had a higher overall clinical note score; however, this difference was not significant (*p* = 0.24).

## Discussion

### Main findings

Evidence suggests that e-learning is as, if not more, effective than traditional education for HSCPs [23, 72]. This review included 21 studies of TEDE for HSCPs and aimed to appraise and synthesise the current evidence from experimental and quasi-experimental research. The review sought to understand if TEDE is beneficial across the outcomes in Kirkpatrick’s model. The included studies contained high-levels of statistical heterogeneity which precluded meta-analysis; therefore, narrative synthesis techniques were helpful to demonstrate findings and programme characteristics across the individual studies. Dealing with heterogeneity and navigating inconsistent use of terminology are particular challenges in the field of virtual learning [20]. Classification of delivery methods and differentiating between ODE and non-networked CBDE reduced complexity; however, the range of pedagogical characteristics within individual programmes hindered the extent to which training effects could be attributed to overarching delivery methods. Therefore, any inference for knowledge attainment following ‘TEDE’ was only made possible due to the quantity of individual studies that provided evidence of training effects. Likewise, most studies that included skills-based outcomes reported positive effects due to the training. Evidence for attitudinal change following TEDE was less compelling; however, it is possible that practitioners’ attitudes can also be influenced through improved knowledge [73]. Fewer studies reported on the higher-level outcomes (i.e., behaviours and results). More research would be useful to understand if TEDE programmes can support wider organisational outcomes, either directly or when mediated through practitioner learning gains. The review included several studies of TEDE in higher education, long-term care, and primary care. There was limited evidence for TEDE in the acute hospital. This will be a priority area for future research that might also consider how TEDE can support complex organisational demands within busy acute care environments.

### Key features for effective TEDE

Surr et al. [27] identified a number of key features that seem to exist in effective dementia training. Passive teaching and learning methods do not reflect educational best practice and recommendations include *active* participation in dementia education. TEDE programmes may achieve this by moving away from self-directed approaches that rely on large amounts of textual information and singular instructional modalities toward more multi-modal programmes that include a rich variety of role-relevant instructional strategies—whether in ODE or CBDE. ODE programmes may enable greater levels of activity by virtue of the Web 2.0 technology that allows learners to collaborate and problem solve together [74]. Surr et al. [27] highlighted this need for *interactivity* in dementia education involving groups of learners and experienced facilitators. Thus, TEDE should aim to meet the individual needs of learners and offer opportunities for collaboration, peer and facilitator support, and group reflective activities [75]. Currently, there is a dearth of evidence on communities of practice and both synchronous and asynchronous communication platforms in ODE. This will be a priority area for future research considering also the recent rapid transition to technology for learning including the accelerated use of chat-based collaboration platforms since COVID-19 [76]. Non-networked CBDE may continue to play a role where learners cannot access online resources, and blended approaches may help to compensate for the pedagogical benefits otherwise derived from interactive learning and Web 2.0. Three studies included blended approaches [52, 66, 68]. One study did not provide evidence for knowledge gains following this approach; however, the training programme was of relatively short duration [68]. Duration of engagement is another important factor in effective dementia education. The total duration of dementia education is relevant as it is likely to influence the training *effects* [27]. It is less clear if TEDE offers any advantages in terms of time *efficiency* over traditional methods for dementia education—as both delivery methods are highly context-specific and likely to be influenced by specific pedagogical characteristics and course design [77]. Four studies included evidence for outcome effects over time [52, 55, 62, 68] albeit inference towards the sustainability of training effects was limited due to inconsistent findings between the studies. It is also important that dementia education has relevance to the role and the experience of learners [27]. The current review suggests that this applies, not only to educational content, but to instructional strategies and modes of information delivery in TEDE programmes. For instance, case-based learning was widely applicable across practice and higher educational settings, whereas practice and problem-based learning were described only in the primary care context [62, 66]. Furthermore, studies from long-term care highlighted the need for simplified modes of information delivery [17, 58, 59]. Future research might consider how technology can be harnessed to respond to the specific learning requirements of individuals or groups by exploring adaptive learning technologies for tailored programmes that include material and instructional strategies that are most relevant [78].

### Methodological issues in TEDE research

Despite debate as to whether evidence hierarchies are appropriate frameworks to judge the quality of educational research [79], benefits of randomised trials and controlled studies have been described in similar reviews of TEDE [28]. Reviewers of traditional dementia education endorse randomised trials when considering intervention effectiveness [11]; others highlight challenges associated with randomised trials in healthcare settings [80]; or suggest integrating qualitative methods to deal with complex educational interventions [81]. MMAT and MERSQI both included methodological quality judgments based on study design. MMAT highlighted significant confounding bias. Extraneous variables can be problematic in educational research, even using the ‘gold standard’ randomised trial [82]. MERSQI attributes higher scores to randomised trials, which helps to address issues of selection bias; however, it is noteworthy that control of confounders is not a specific quality indicator. We do not suggest that confounders can be overlooked in TEDE research; however, where study designs do not have robust strategies to circumvent bias arising from confounders, researchers might place a greater emphasis on the *acknowledgement* of potential confounders and apply controls where it is practical. MMAT appraisal resulted in high levels of uncertainty across many of the other quality domains. This inferred methodological limitations in the primary studies and possible limitations of MMAT to the TEDE research context. For instance, participant representativeness was frequently judged to be unclear; however; this was often as a result of non-probability sampling and intentionally pragmatic methods using accessible learner cohorts. In this context, quality judgements may improve if researchers provide clearer justification for pragmatic methods and acknowledge the associated study limitations including the generalisability of findings. Issues from outcome measurement were covered in both MMAT and MERSQI. For study results to be credible, assessment instruments must be both reliable and valid [83]. In TEDE research, there may be a greater need for validation *in context* for optimal cohesion between the instructional content and the outcomes measured. In general, MMAT allowed for the appraisal of various study designs common in educational research but may have lacked the specificity required to appraise diverse and often pragmatic educational research methods. MERSQI was a valuable complementary tool as it omitted many issues common in educational research and put additional emphasis on the number of institutions studied, complexity of data analysis techniques, and key educational outcomes—which were well-aligned with Kirkpatrick’s model.

### Dealing with causality and complexity

The main advantage of including experimental and quasi-experimental studies in the review is capacity to establish causal inferences between the TEDE programme and the outcomes measured. However, overall conclusions must be treated with caution. The technology alone cannot influence the training effects; rather, it is the *pedagogy* that the technology enables and supports—and pedagogy differs widely between training programmes [74]. Even where similarities exist (e.g., use of case-based instruction, videos, textual information), the approach is likely to be influenced by intrinsic factors relating to content, quality, and duration, as well as extrinsic factors such as learner engagement. Therefore, these pedagogical strategies need to be considered as layered elements that are nested within programmes and require methods of evaluation that can illicit their specific role and function [84]. Where multiple causal elements exist, methods of evaluation become more complicated, yet it is nonetheless important that these elements are considered in evaluation processes. One way to address this complexity is to employ principles from programme theory which refers to a variety of ways of developing causal models that can link various programme inputs and elements to the intended outcomes [85]. Each element can then be identified and addressed through the pragmatic integration of the most relevant quantitative *or* qualitative research methods; thereby, gaining a deeper understanding of the more nuanced elements, which, when brought back together, are likely to result in a more meaningful understanding of the ‘whole’. Scerri et al. [24] suggested a more inclusive approach to dementia education research towards a richer understanding of complexity and enhanced ecological validity. It is clear that interventional complexity and ‘real world’ influence cannot be easily eliminated in TEDE research. Future research might therefore shift focus now from internal validity to the most relevant research design typologies that can achieve a more nuanced understanding of these complex educational programmes and context [79].

## Limitations

There are several limitations of this review. Firstly, all attempts were made to conduct a comprehensive literature search and study screening process; however, two references entitled ‘resources’ could not be located and were excluded at the screening stage. Furthermore, this review only included studies available in the English language. Most titles and abstracts and all full texts were assessed for eligibility by one reviewer which was necessary due to resource limitations. Similarly, one person completed data extraction which may have increased the risk of errors [86]. Quality appraisal was also completed by one reviewer; however, it is worth noting that MERSQI focuses on design issues and is quite objective [50]. It is also worth noting that the overall quality of studies was judged to be ‘moderate’—which is consistent with other reviews of TEDE [28]. Second reviewers appraised a proportion of studies using MMAT which mitigated against bias where more subjective judgments were involved; however, the application of MMAT to this review context required additional justification as it is traditionally used in systematic mixed studies reviews. It is possible that the exclusion of studies published before 2005 disproportionately limited the evidence for CBDE compared to ODE. This was a pragmatic decision that balanced the need to segregate the two approaches (i.e., to address potential limitations where there may be sub-optimal technological infrastructure for ODE) whilst maintaining a contemporary focus. It is also important to note that CBDE may be reproducible as ODE and vice versa. The review included experimental and quasi-experimental studies and excluded descriptive narrative and survey evaluations of TEDE. This limited the extent to which outcomes based on Kirkpatrick’s level 1 (reactions) featured in the review. This limitation was anticipated as participant satisfaction levels are typically evaluated as learner feedback *at the end* of training programmes which can support future programme development [33, 87]. Satisfaction levels before and after training were reported in two studies of CBDE; however, these related to general concepts such as motivation for learning [63] and comfort with computer-based training [65]. It is not possible to determine pre-test levels of satisfaction with a specific training programme. Post-test comparisons of participant satisfaction between intervention and comparator groups may be achievable; however, the data available did not support this type of analysis. It is important to highlight that many primary studies included complementary reports of participant satisfaction using appropriate descriptive methods of evaluation. Training effects were more appropriately described as improvements in learning gains (knowledge, attitudes, and skills), practitioner behaviours, and wider results due to the training. However, effects were most frequently assumed from controlled studies involving a ‘no training’ control arm, or from before-after studies—where the before aspect could be considered a ‘no training’ state. Few studies compared the relative effectiveness of TEDE compared to alternative delivery methods (e.g., traditional education). This will also be a priority focus for future research. Finally, the review did not provide an exhaustive inventory of TEDE programmes as several innovative practices may have been identified in descriptive research that did not satisfy the study selection criteria. Future evidence syntheses that include methods to incorporate these rich sources of information will be crucial to better understand how technology can enable dementia education for the health and social care workforce.

## Conclusion

The current evidence provides several examples where TEDE is beneficial to practitioner development on emotional, as well as intellectual levels, which is crucial for person-centred dementia care. The evidence highlights a need for more emphasis on the teaching and learning methods within TEDE and the requirements of specific learning communities. Establishing innovative active and interactive learning strategies will be integral to the design and development of future training programmes. Future evaluations might explore the relative effectiveness of TEDE compared to traditional dementia education and employ contextually relevant research methods that have capacity to address the challenges presented by these complex educational programmes and multi-component characteristics.

## Supplementary Information

**Additional file 1.** PRISMA Checklist.

**Additional file 2.** Search Strategy.

**Additional file 3.** Sample Data Extraction Form.

**Additional file 4.** Characteristics of Included Studies.

**Additional file 5.** Excluded Studies.

**Additional file 6.** MERSQI Scores (Primary Studies).

## Data Availability

Not applicable.

## Abbreviations

BPS: Branching path simulation
CBDE: Computer-based dementia education
FU: Follow-up
HSCP: Health and social care practitioner
MCQ: Multiple choice question
MERSQI: Medical Education Research Study Quality Instrument
MMAT: Mixed methods appraisal tool
ODE: Online dementia education
OSCE: Objective structured clinical examination
PRISMA: Preferred reporting items for systematic review and meta-analysis
PROSPERO: International prospective register of systematic reviews
SD: Standard deviation
TEDE: Technology-enabled dementia education
