# Supplementary material for: Establishing the effectiveness of technology-enabled dementia education for health and social care practitioners: a systematic review
Source: Syst Rev. 2021 Sep 21;10:252. doi: 10.1186/s13643-021-01781-8 (PMC8452826; doi:10.1186/s13643-021-01781-8)
Supplement: Supplementary file 3 — Additional file 3. Sample Data Extraction Form. [file 13643_2021_1781_MOESM3_ESM.docx]

**Additional file 3. Sample Data Extraction Form**

**Author & Title:**

| *[Insert additional comments]*   \| MERSQI Domain \| MERSQI Item \| **Score** \| **Max Score**  **Item** \| **Domain**  **Score** \| \| --- \| --- \| --- \| --- \| --- \| \| Study design \| 1. Study design \| \|  \|  \| \| Single group cross‐sectional or single group post-test only \| 1 \| \| Single group pre-test & post-test \| 1.5 \|  \| \| Nonrandomized, 2 groups \| 2 \|  \| \| Randomized controlled trial \| 3 \|  \| \| Sampling \| 2. Institutions studied \| \|  \|  \| \| 1 \| 0.5 \| \| 2 \| 1 \| \| 3 \| 1.5 \| \| 3. Response rate, % \| \| \| Not applicable \|  \| \| <50 or not reported \| 0.5 \| \| 50‐74 \| 1 \| \| >75 \| 1.5 \| \| Type of data \| 4. Type of data \| \|  \|  \| \| Assessment by participants \| 1 \| \| Objective measurement \| 3 \| \| Validity of evaluation instrument \| 5. Internal structure \| \|  \|  \| \| Not applicable \|  \| \| Not reported \| 0 \| \| Reported \| 1 \| \| 6. Content \| \|  \| \| Not applicable \|  \| \| Not reported \| 0 \| \| Reported \| 1 \| \| 7. Relationships to other variables \| \|  \| \| Not applicable \|  \| \| Not reported \| 0 \| \| Reported \| 1 \| \| Data analysis \| 8. Appropriateness of analysis \| \|  \|  \| \| Inappropriate for study design or type of data \| 0 \| \| Appropriate for study design, type of data \| 1 \| \| 9. Complexity of analysis \| \|  \| \| Descriptive analysis only \| 1 \| \| Beyond descriptive analysis \| 2 \| \| Outcomes \| 10. Outcome \| \|  \|  \| \| Satisfaction, attitudes, perceptions, opinions, general facts \| 1 \| \| Knowledge, skills \| 1.5 \| \| Behaviours \| 2 \| \| Patient/health care outcome \| 3 \| \| Total Score \| \|  \|  \|  \|  \| MMAT *[Insert Study Design Category]* \| Authors' judgement \| Support for judgement \| \| --- \| --- \| --- \| \| *Insert relevant methodological quality criteria* \|  \|  \| \| *Insert relevant methodological quality criteria* \|  \|  \| \| *Insert relevant methodological quality criteria* \|  \|  \| \| *Insert relevant methodological quality criteria* \|  \|  \| \| *Insert relevant methodological quality criteria* \|  \|  \|   *[Insert additional comments]* | |
| --- | --- | --- | --- | --- | --- | --- | --- | --- | --- | --- | --- | --- | --- | --- | --- | --- | --- | --- | --- | --- | --- | --- | --- | --- | --- | --- | --- | --- | --- | --- | --- | --- | --- | --- | --- | --- | --- | --- | --- | --- | --- | --- | --- | --- | --- | --- | --- | --- | --- | --- | --- | --- | --- | --- | --- | --- | --- | --- | --- | --- | --- | --- | --- | --- | --- | --- | --- | --- | --- | --- | --- | --- | --- | --- | --- | --- | --- | --- | --- | --- | --- | --- | --- | --- | --- | --- | --- | --- | --- | --- | --- | --- | --- | --- | --- | --- | --- | --- | --- | --- | --- | --- | --- | --- | --- | --- | --- | --- | --- | --- | --- | --- | --- | --- | --- | --- | --- | --- | --- | --- | --- | --- | --- | --- | --- | --- | --- | --- | --- | --- | --- | --- | --- |
| Citation |  |
| Study Design |  |
| Aims |  |
| Country |  |
| Ethics |  |
| Participant Characteristics |  |
| Setting |  |
| Participant Demographics |  |
| Results of Quality Assessment |  |
| Sampling Approach |  |
| Inclusion Criteria |  |
| Exclusion Criteria |  |
| Data Collection Instruments |  |
| Internal Validity/ Reliability |  |
| Data Analysis Methods |  |
| Power Calculation |  |
| Response Rate / Outcome Data |  |
| Intervention |  |
| Educational Content |  |
| Technical Characteristics |  |
| Pedagogical Approach |  |
| Duration |  |
| Educational/ Pedagogical Theories |  |
| Comparator/ Control Group |  |
| Learner Satisfaction |  |
| Knowledge |  |
| Skills |  |
| Attitudes |  |
| Behaviours |  |
| Results |  |
| Educator experience |  |
| Functionality |  |
| Technical Support |  |
| Usability (PU/ PEU) |  |
| Cost-effectiveness |  |
| Attrition |  |
| Opportunity for reflection |  |
| Curricular Basis (Dementia) |  |
