# Supplementary material for: Establishing the effectiveness of technology-enabled dementia education for health and social care practitioners: a systematic review
Source: Syst Rev. 2021 Sep 21;10:252. doi: 10.1186/s13643-021-01781-8 (PMC8452826; doi:10.1186/s13643-021-01781-8)
Supplement: Supplementary file 6 — Additional file 6. MERSQI Scores (Primary Studies). [file 13643_2021_1781_MOESM6_ESM.docx]

**Additional file 6a. MERSQI Scores (Primary Studies)**

| First Author (Year) | Study Design | Sampling | | Type of Data | Validity of evaluation instrument | | | Data Analysis | | Outcomes | Total |
| --- | --- | --- | --- | --- | --- | --- | --- | --- | --- | --- | --- |
|  |  | Institutions studied | Response rate, % |  | Internal structure | Content | Relationships to other variables | Appropriate analysis | Complexity of analysis |  |  |
| Tsai (2018) | 2 | 1 | 1.5 | 3 | 1 | 1 | 0 | 1 | 2 | 3 | 15.5 |
| Tomaz (2015) | 2 | 1.5^1^ | 1.5 | 3 | 1 | 1 | 0 | 1 | 2 | 1.5 | 14.5 |
| Hobday et al. (2010) | 1.5 | 1.5 | 1.5 | 3 | 1 | 1 | 0 | 1 | 2 | 1.5 | 14.0 |
| Downs (2006) | 3 | 1.5 | 1^2^ | 3 | 0 | 0 | 0 | 1 | 2 | 2 | 13.5 |
| Hobday, Savik, and Gaugler (2010) | 1.5 | 1.5 | 1 | 3 | 1 | 1 | 0 | 1 | 2 | 1.5 | 13.5 |
| Chao (2016) | 1.5 | 1.5^3^ | 1.5 | 1 | 1 | 1 | 0 | 1 | 2 | 3 | 13.5 |
| Cobbet (2016) | 2 | 0.5^4^ | 1.5^5^ | 3 | 1 | 1 | 0 | 1 | 2 | 1.5 | 13.5 |
| Kimzey (2016) | 2 | 0.5 | 1.5 | 3 | 1 | 0 | 1 | 1 | 2 | 1.5 | 13.5 |
| Matsumura (2018) | 2 | 0.5 | 1.5 | 3 | 1 | 1 | 0 | 1 | 2 | 1.5 | 13.5 |
| Luconi (2008)^6^ | 1.5 | 1.5 | 1.5 | 3 | 0 | 1 | 0 | 1 | 2 | 1.5 | 13.0 |
| Hobday (2017) | 1.5 | 1.5 | 1.5 | 3 | 0 | 1 | 0 | 1 | 2 | 1.5 | 13.0 |
| Vollmar (2010) | 3 | 1.5 | 0.5^7^ | 3 | 0 | 0 | 0 | 1 | 2 | 1.5 | 12.5 |
| Westmoreland (2010) | 3 | 0.5 | 0.5^8^ | 3 | 0 | 0 | 0 | 1 | 2 | 2 | 12.0 |
| Helms (2009) | 2 | 0.5 | 1.5 | 3 | 0 | 0 | 0 | 1 | 2 | 1.5 | 11.5 |
| Rababa (2020) | 2 | 0.5 | 1.5 | 1 | 1 | 1 | 0 | 1 | 2 | 1.5 | 11.5 |
| De Witt Jansen (2018) | 1.5 | 1.5 | 1.5 | 1 | 0 | 1 | 0 | 1 | 2 | 1.5 | 11.0 |
| Bentley (2019) | 1.5 | 1.5 | 1.5 | 3 | 0^9^ | 0 | 0 | 1 | 1 | 1.5 | 11.0 |
| Irvine (2007) | 3 | 0.5^10^ | 1.5 | 1 | 0 | 0 | 0 | 1 | 2 | 1.5 | 10.5 |
| Irvine 2013) | 1.5 | 0.5 | 1.5 | 1 | 1 | 0 | 0 | 1 | 2 | 1.5 | 10.0 |
| Jones (2016) | 1.5 | 1.5 | 0.5^11^ | 1 | 1 | 0 | 0 | 1 | 2 | 1.5 | 10.0 |
| Ruiz (2006) | 1.5 | 0.5 | 1.5^12^ | 1 | 0 | 0 | 0 | 1 | 2 | 1.5 | 9.0 |
| Mean | **1.95** | **1.05** | **1.31** | **2.33** | **0.52** | **0.52** | **0.05** | **1.00** | **1.95** | **1.69** | **12.4** |
| Mode | **1.5** | **1.5** | **1.5** | **3** | **1** | **1** | **0** | **1** | **2** | **1.5** |  |

1 Assumes that physicians were from multiple family health teams

2 Based on cases identified and total valid for analysis

3 Nurses were recruited from a variety of long-term care facilities

4 Geographically separate campuses, same institution

5 Based on ‘final sample’

6 The assessment relates to the pre- and post-test objective measures and may not be relevant to the wider descriptive case study.

7 Considers all participant groups from t_0_-t_2_

8 Reflects variability in response rates which in some cases is less than 50%

9 DKAS and GPACS-D are reported to have been previously validated with citations provided. However, specific validity indicators (i.e. internal structure / content) were not reported by study authors.

10 Multi-institutions are not clear/relevant

11 Numbers completing pre- and post-tests not explicitly reported (*with 4 weeks between tests*)

12 Assumes all 38 participants completed post-tests (*as intervention was completed over 2 days to “include all the participants”*)

**Additional file 6b. MERSQI Decision-Making Tool (for the review context)**

| **Study design** | Choose the relevant study design.   - Choose ‘single group pre- and post-test’ if 1 group or if > 1 group where pre- and post-tests are not being compared - Choose ‘nonrandomized 2 groups’ if 2 or more (nonrandomized) groups are being compared |
| --- | --- |
| **Institutions studied** | Include health / social care settings and educational establishments as institutions.  Consider that participants may be sampled from multiple institutions.  Choose the relevant number of institutions.   - Score 1.5 if multiple settings (>2) are involved and the exact number is not specified - Consider 0.5 if the number of settings is not reported or is unclear. |
| **Response rate** | Multiple / control groups:   - Consider response rate as the relationship between the initial participants taking part in interventions and completing final evaluation.   Single group pre- and post-tests:   - Consider response rate as the relationship between participants completing pre-tests compared to post-tests. Consider not reported if pre- and post-test participant numbers are not clearly reported. |
| **Type of data** | Only include measures that relate to the review outcomes. Consider test-based outcome measures (i.e. MCQ, right or wrong answers) as being ‘objective’. Participant perceptions / beliefs (i.e. Likert Scale responses) are considered subjective judgements. Answer according to the majority approach where multiple measures are involved. Choose ‘objective’ if there is no majority. |
| **Validity of evaluation instrument** | Only include measures that relate to review outcomes. Consider in relation to each specific validity indicator (i.e. internal structure / content) and respond favourably if robust examples of validity assessment are specified (consider previously validated tools and discrete reports of alpha statistics).   - Consider content validation where strategies are reported to ensure appropriate content representation (this may included analysis by experts) - Consider that internal structure is often reported as measures of internal consistency reliability / factor analysis - Consider relationship to other variables as being either positive (convergent or predictive) or negative (divergent or discriminant) - where reported (1)   In studies reporting multiple outcome measurements, answer ‘reported’ for each specific validity indicator where there is evidence of reporting for the majority of outcome measures. Choose ‘reported’ if there is no majority. Choose ‘not reported’ where there is reporting in the minority of outcome measures. |
| **Data Analysis** | Choose the appropriateness of data analysis. |
| **Outcome** | Choose the highest level of outcome reported. |

1 Beckman TJ, Cook DA, Mandrekar JN. What is the validity evidence for assessments of clinical teaching?. J Gen Intern Med. 2005;20(12):1159-1164.

Note: the decision making tool was developed to promote consistent scoring between studies and is specific to this review context. It is not indended for use in any other context.

**Additional file 6c. MERSQI Domains and Scoring Criteria**

| MERSQI Domain | MERSQI Item | **Score** | **Max Score**  **Item** | **Domain**  **Score** |
| --- | --- | --- | --- | --- |
| Study design | 1. Study design | |  |  |
|  | Single group cross‐sectional or single group post-test only | 1 |  |  |
|  | Single group pre-test & post-test | 1.5 |  |  |
|  | Nonrandomized, 2 groups | 2 |  |  |
|  | Randomized controlled trial | 3 |  |  |
| Sampling | 2. Institutions studied | |  |  |
|  | 1 | 0.5 |  |  |
|  | 2 | 1 |  |  |
|  | 3 | 1.5 |  |  |
|  | 3. Response rate, % | |  |  |
|  | Not applicable |  |  |  |
|  | <50 or not reported | 0.5 |  |  |
|  | 50‐74 | 1 |  |  |
|  | >75 | 1.5 |  |  |
| Type of data | 4. Type of data | |  |  |
|  | Assessment by participants | 1 |  |  |
|  | Objective measurement | 3 |  |  |
| Validity of evaluation instrument | 5. Internal structure | |  |  |
|  | Not applicable |  |  |  |
|  | Not reported | 0 |  |  |
|  | Reported | 1 |  |  |
|  | 6. Content | |  |  |
|  | Not applicable |  |  |  |
|  | Not reported | 0 |  |  |
|  | Reported | 1 |  |  |
|  | 7. Relationships to other variables | |  |  |
|  | Not applicable |  |  |  |
|  | Not reported | 0 |  |  |
|  | Reported | 1 |  |  |
| Data analysis | 8. Appropriateness of analysis | |  |  |
|  | Inappropriate for study design or type of data | 0 |  |  |
|  | Appropriate for study design, type of data | 1 |  |  |
|  | 9. Complexity of analysis | |  |  |
|  | Descriptive analysis only | 1 |  |  |
|  | Beyond descriptive analysis | 2 |  |  |
| Outcomes | 10. Outcome | |  |  |
|  | Satisfaction, attitudes, perceptions, opinions, general facts | 1 |  |  |
|  | Knowledge, skills | 1.5 |  |  |
|  | Behaviours | 2 |  |  |
|  | Patient/health care outcome | 3 |  |  |
| Total Score | |  |  |  |
